# Supplementary material for: Is yearly interferon gamma release assay latent tuberculosis infection screening warranted among patients with rheumatological diseases on disease-modifying drugs in non-endemic settings?
Source: PLoS One. 2024 Jul 3;19(7):e0306337. doi: 10.1371/journal.pone.0306337 (PMC11221665; doi:10.1371/journal.pone.0306337)
Supplement: S1 Table — Disease-modifying antirheumatic drugs (DMARDs) were grouped by mechanism of action. (DOCX) [file pone.0306337.s001.docx]

**Supplementary Materials for:**

**Is yearly interferon gamma release assay latent tuberculosis infection screening warranted among patients with rheumatological diseases on disease-modifying drugs in non-endemic settings?**

**S1 Table**. Disease Modifying Anti-Rheumatic Drugs (DMARD) Categories

| **DMARD categories** | **DMARD** |
| --- | --- |
| TNF-alpha inhibitor | golimumab |
|  | Certolizumab |
|  | Etarnacept |
|  | Adalimumab |
|  | Infliximab |
| IL-6 and IL-6 receptor blockers | Tocilizumab |
|  | Sarilumumab |
| T-cell co-stimulation inhibitor | Abatacept |
| IL-1 antagonists | Anakinra |
| B-cell depleting agents or Blys/BAFF inhibitor | Rituximab |
|  | Belimumab |
| IL-17A inhibitors | Secukinumab |
|  | Ixekizumab |
| IL-12/23 axis blockade | Risankizumab |
|  | Tildrakizumab |
|  | Guselkumab |
|  | Ustekinumab |
| Cyclophosphamide |  |
| JAK inhibitors | Tofacitinib |
|  | Upadacitinib |
|  | Baricitinib |
| Vedolizumab |  |

*****Disease-modifying antirheumatic drugs (DMARDs) were grouped by mechanism of action
