# Supplementary material for: Is yearly interferon gamma release assay latent tuberculosis infection screening warranted among patients with rheumatological diseases on disease-modifying drugs in non-endemic settings?
Source: PLoS One. 2024 Jul 3;19(7):e0306337. doi: 10.1371/journal.pone.0306337 (PMC11221665; doi:10.1371/journal.pone.0306337)
Supplement: S2 Table — (DOCX) [file pone.0306337.s002.docx]

**Supplementary Materials for:**

**Is yearly interferon gamma release assay latent tuberculosis infection screening warranted among patients with rheumatological diseases on disease-modifying drugs in non-endemic settings?**

**S2 Table** Rheumatic Disease Categories

| **Rheumatic Disease Categories** | **Rheumatic Diseases** |
| --- | --- |
| Inflammatory Arthritis | Rheumatoid arthritis |
|  | Psoriasis/Psoriatic arthritis |
|  | Ankylosing spondylitis |
|  | Seronegative inflammatory arthritis |
|  | Enteropathic arthritis or inflammatory bowel disease-associated arthritis |
|  | Juvenile idiopathic arthritis |
| Connective tissue disease | Systemic lupus erythematosus |
|  | Sjogren’s disease |
|  | Systemic Sclerosis |
|  | Mixed Connective tissue disease |
|  | Undifferentiated connective tissue disease |
|  | Connective tissue disease overlap syndrome |
| Vasculitis | Giant cell arteritis |
|  | Takayasu arteritis |
|  | ANCA-associated vasculitis (including Granulomatosis with polyangiitis, Microscopic polyangiitis, and Eosinophilic granulomatosis with polyangiitis) |
|  | Pauci-immune vasculitis |
|  | Leukocytoclastic vasculitis |
|  | Cerebral vasculitis |
|  | Cryoglobulinemic vasculitis |
|  | Urticarial vasculitis |
| Idiopathic inflammatory myositis | Dermatomyositis or polymyositis |
|  | Antisynthetase syndrome |
| Polymyalgia rheumatica |  |
| Miscellaneous diseases | Still’s disease |
|  | Sarcoidosis |
|  | Behcet’s disease |
|  | Hidradenitis suppuritiva |
|  | Antiphospholipid syndrome |
