# Supplementary material for: Is yearly interferon gamma release assay latent tuberculosis infection screening warranted among patients with rheumatological diseases on disease-modifying drugs in non-endemic settings?
Source: PLoS One. 2024 Jul 3;19(7):e0306337. doi: 10.1371/journal.pone.0306337 (PMC11221665; doi:10.1371/journal.pone.0306337)
Supplement: S3 Table — (DOCX) [file pone.0306337.s003.docx]

**Supplementary Materials for:**

**Is yearly interferon gamma release assay latent tuberculosis infection screening warranted among patients with rheumatological diseases on disease-modifying drugs in non-endemic settings?**

**S3 Table.** TB history

|  | N | % |
| --- | --- | --- |
| History of LTB | 15 | 4.4 |
| 2017 QTF (n=118)  Negative  Positive  Indeterminate | 113  2  3 | 95.8  1.7  2.5 |
| 2018 QTF (n=153)  Negative  Positive  Indeterminate | 145  2  6 | 94.8  1.3  3.9 |
| 2019 QTF (n=167)  Negative  Positive  Indeterminate | 155  3  9 | 92.8  1.8  5.4 |
| 2020 QTF (n=236)  Negative  Positive  Indeterminate | 224  3  9 | 94.9  1.3  3.8 |
| 2021 QTF (n=263)  Negative  Positive  Indeterminate | 257  4  2 | 97.7  1.5  0.8 |
| Any positive QTF | 9 | 2.6 |
| TB predisposing factors  Smoking use  Heavy alcohol use  Substance use  Diabetes  HIV | 14  5  3  49  2 | 4.1  1.5  0.9  14.4  0.6 |
