# Supplementary material for: Is yearly interferon gamma release assay latent tuberculosis infection screening warranted among patients with rheumatological diseases on disease-modifying drugs in non-endemic settings?
Source: PLoS One. 2024 Jul 3;19(7):e0306337. doi: 10.1371/journal.pone.0306337 (PMC11221665; doi:10.1371/journal.pone.0306337)
Supplement: S4 Table — (DOCX) [file pone.0306337.s004.docx]

**Supplementary Materials for:**

**Is yearly interferon gamma release assay latent tuberculosis infection screening warranted among patients with rheumatological diseases on disease-modifying drugs in non-endemic settings?**

**S4 Table.** Characteristics of patients with conversion to positive QTF

| Patient | Age | Sex | Rheumatological diagnosis | DMARD | LTBI diagnosis | Treatment of LTBI | TB risk factors |
| --- | --- | --- | --- | --- | --- | --- | --- |
| A | 35 | Female | Behcet’s disease | Tocilizumab | Repeat QFT was negative | N/A | N/A |
| B | 72 | Male | Psoriatic arthritis | Secukinumab | Repeat QFT was negative | N/A | N/A |
| C | 58 | Female | Rheumatoid arthritis | Etanercept | Previous LTBI | Treated with INH for 9 months 11 years ago | Born in Peru |
| D | 53 | Female | Psoriatic arthritis | Secukinumab | New LTBI | Treated with RIF for 4 months, 3 years ago | Former smoker, works in healthcare |
