# Supplementary material for: Is yearly interferon gamma release assay latent tuberculosis infection screening warranted among patients with rheumatological diseases on disease-modifying drugs in non-endemic settings?
Source: PLoS One. 2024 Jul 3;19(7):e0306337. doi: 10.1371/journal.pone.0306337 (PMC11221665; doi:10.1371/journal.pone.0306337)

**Supplementary Materials for:**

**Is yearly interferon gamma release assay latent tuberculosis infection screening warranted among patients with rheumatological diseases on disease-modifying drugs in non-endemic settings?**

**S2 Fig. Suggested LTBI screening approach by TB epidemiologic risk factors.**


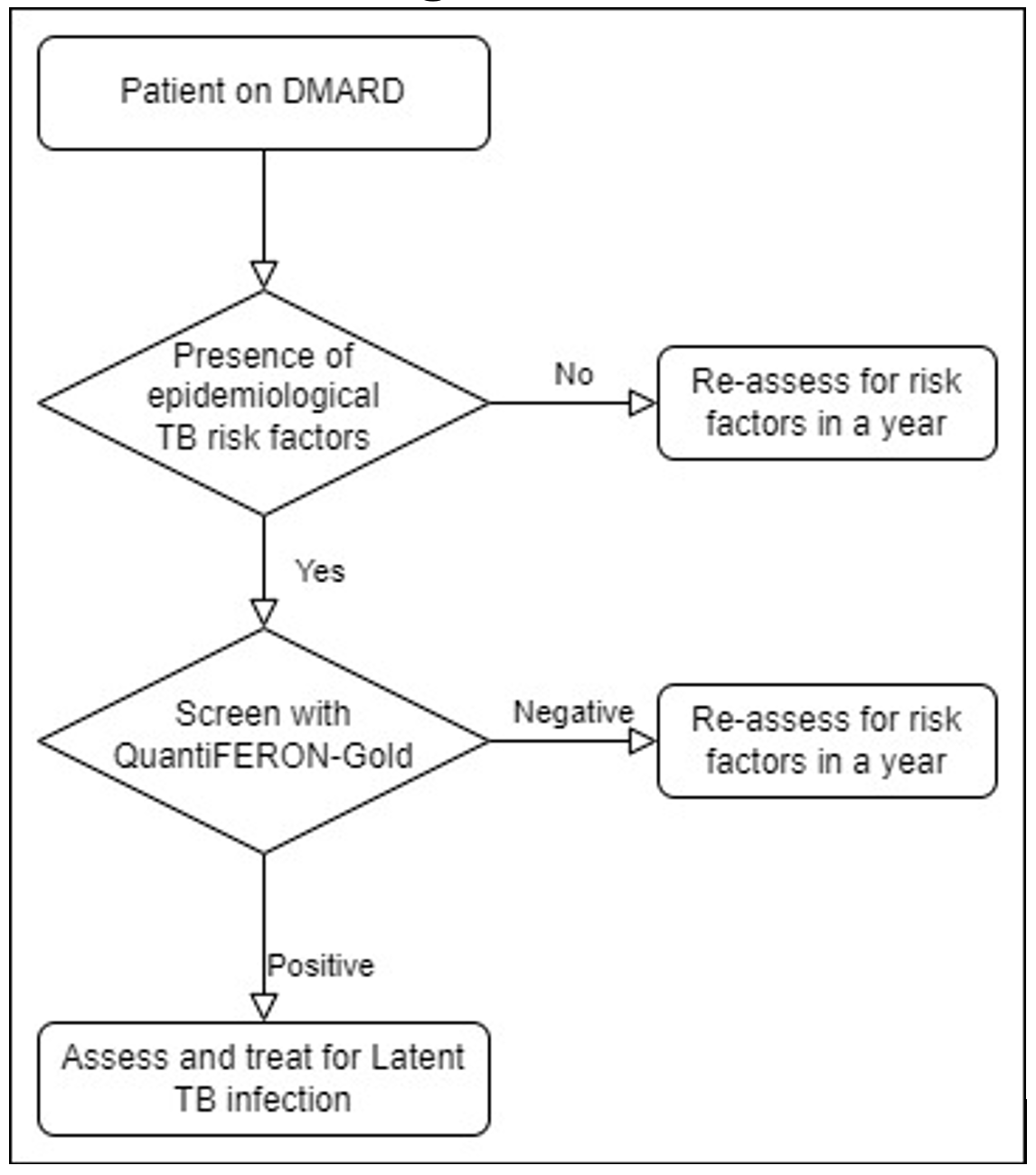

Supplement: S2 Fig — (DOCX) [file pone.0306337.s006.docx]
