# Supplementary material for: Is yearly interferon gamma release assay latent tuberculosis infection screening warranted among patients with rheumatological diseases on disease-modifying drugs in non-endemic settings?
Source: PLoS One. 2024 Jul 3;19(7):e0306337. doi: 10.1371/journal.pone.0306337 (PMC11221665; doi:10.1371/journal.pone.0306337)
Supplement: Attachment — Submitted filename: Cover Letter Response May 18.docx [file pone.0306337.s008.docx]

May 18, 2024

To the Editor and Reviewers

We are truly grateful for the review and comments of the reviewers. We revised the paper to clarify the comments of the reviewer, which we think also improved the paper’s clarity in describing the results.

Below is our response to the reviewer’s comment ( in blue) :

Review Comments to the Author

Reviewer #2: The authors have addressed all comments except one:

Reviewer #2: Could you please specify if the 18 patients with a positive IGRA at first screening were eligible for preventive therapy and, if not, why?

**We want to address a crucial point raised by Reviewer 2. Of the 339 patients who tested at least twice during the study period, only 18 had documented yearly testing as described in the demographics section ( line 101, page 6). In 2017 (the start of the study), only 2 tested positive for IGRA, and both had prior documented LTBI treatment (page 7, line 115-116). The abstract was also revised to make it clear of results at baseline.**

**We revised the tuberculosis history and IGRA results to clarify the circumstances of the positive testing. We created a subsection of those who converted from negative to positive tests and the course thereafter.**

**All revisions were highlighted in yellow. Thank you again for the comment.**

Comment from review #2: The second point which needs clarification is the policy regarding the 18 patients with a positive test result at initial screening, before the implementation of biological treatment. The authors should indicate if these patients were eligible for a preventive treatment and what are the criteria for it.

**Please see the response to the prior comment.**

Sincerely,

Maricar Malinis

Carlos Fopianno Palacios
